# The relationship between sports participation and bullying in elementary schools: the mediating role of mental toughness

**DOI:** 10.3389/fpsyg.2025.1463705

**Published:** 2025-04-09

**Authors:** Changhao Ma, Mingze Gao, Ziyang Qi, Wen Che, Shenghua Qi

**Affiliations:** ^1^College of Physical Education, University of Jinan, Jinan, China; ^2^School of Education and Psychology, University of Jinan, Jinan, China

**Keywords:** sports participation, mental toughness, school bullying, elementary school, physical and mental health

## Abstract

**Purpose:**

Previous research has shown a correlation between sports participation and bullying in schools. However, less attention has been paid to the factors influencing children’s perpetrated bullying and victimized. Therefore, this study constructed a model of the role of influencing factors between sports participation, children’s mental toughness, and school bullying to explore the link between sports participation and mental toughness on children’s perpetrated bullying and victimized.

**Methods:**

A sample of 861 students was randomly selected from five elementary schools in Shandong Province, China. After excluding invalid questionnaires with missing answers or consistent responses, 712 questionnaires were collected. The Physical Activity Rating Scale (PARS-3) was selected to assess sports participation, the Mental Toughness Scale to evaluate mental resilience, and the Chinese-adapted version of the Oweis Bullying Questionnaire (OBQ), which was revised by Chinese scholar Zhang Xinwen, to measure bullying experiences. All participants completed maturation scales on sports participation, mental toughness, and school bullying. Data were analyzed in SPSS 24.0, SPSSprocess 4.1, and AMOS 27.

**Results:**

The results of this study are as follows: (1) According to the correlation test results, since *p* < 0.05 or *p* < 0.01, there is a correlation between sports participation and psychological resilience, bullying and being bullied. There is a correlation between mental resilience and bullying, being bullied. There is a correlation between bullying and being bullied. (2) Mental toughness plays a mediating role in the influence of sports participation on the perpetrated bullying and in the influence of sports participation on victimized. (3) In the pathways of sports participation, mental toughness, and school bullying, it suggested that mental toughness played a partial mediating role in this pathway. For the pathways of sports participation, mental toughness, and children’s exposure to bullying. The mental toughness played a full mediating role in this pathway.

**Conclusion:**

It is recommended that early intervention and support be given to promote children’s physical mobility and increase their own levels of sports participation and mental toughness, thereby reducing the likelihood of children committing or experiencing bullying in schools and improving their physical and mental health.

## 1 Introduction

Bullying in schools is prevalent globally, and in 2016, a survey of more than 100,000 primary and secondary school students in China showed that 4.7% of the students said they were often bullied by others, while 28.66% said they were occasionally bullied by others (Yao, 2017). UNESCO released its latest report on the state of bullying in schools around the world on 17 January in Seoul, South Korea. According to the report, about 246 million children and adolescents are exposed to school violence and bullying each year, which harms their physical and mental health, and all children and adolescents are at risk of school violence and bullying (UNESCO, 2017). The frequent occurrence of bullying in schools has attracted widespread attention from all sectors of society, especially in March 2024 in a village in Handan, where a student was brutally murdered in a field after being demanded money by several classmates. The Handan incident also showed that bullying in schools tended to be more pronounced at a younger age (Li et al., 2024; Jiang and Shi, 2024). Bullying is not merely a phenomenon that occurs within schools. Student-on-student bullying refers to incidents that happen within or outside schools and among students. In such cases, one party intentionally or maliciously, once or repeatedly, uses physical, verbal and online means to carry out bullying and insulting behaviors, causing the other party to suffer physical harm, property loss and mental damage (Wang, 2019). In UNESCO’s definition of bullying in schools, bullying by teachers against students is also included (UNESCO, 2017). However, according to the existing practical problems and hotspots of concern, the research on school bullying in this paper mainly focuses on physical and verbal bullying among students, and does not include cyber bullying.

School bullying can be seen as a form of child abuse: i.e., peer abuse (Dawkins, 1995). Bullying behavior is often related to the student’s own physical and mental state, often showing dissatisfaction with the school, and the bullied is often a better student or a lonely student (Forero et al., 1999). The students who commit bullying often have a sense of satisfaction in their hearts, which will have a negative impact on their future development (Chapell et al., 2006). Bullying can destroy the cooperative relationship between students (Wang and Chen, 2024). Compared with those who have not been bullied, the victims of bullying often experience physical and psychological symptoms such as decreased academic performance, decreased sleep quality, sadness or headaches (Williams et al., 1996). Research suggests that intervening in school bullying during primary school stage can significantly reduce the risk of bullying among students (Hesapçioglu and Yesilova, 2015; Tian et al., 2023). Therefore, it is particularly important to enhance students’ emotional output and improve their mental health to prevent and reduce bullying in schools.

### 1.1 Sports participation and school bullying

Participation in sports includes things directly or indirectly related to sports (Brown, 2000). It refers to the activities where individuals or groups, for their own needs, purposefully employ sports methods and means to achieve sports goals (Liu, 1993). At this stage, the impact of sports participation on bullying in schools is mainly divided into two aspects: on the one hand, the impact of sports participation on bullying; on the other hand, the impact of sports participation on being bullied. Studies have found that when children are in conflict with each other, weaker children are more likely to become the weaker party because they are not physically dominant, and thus are bullied by better children (Ji et al., 2004). However, the study from Hallal et al. (2006) has found that increased levels of sports participation can help to improve their own health and reduce the incidence of disease, thus improving their physical fitness. This can reduce bullying among children effectively. Increased participation level in sports not only improves children’s physical fitness, but also increases communication with classmates. Research has shown that for children in the upper primary grades, their defenses against bullying are positively correlated with emotional regulation and emotional control (Jenkins et al., 2018). This further suggests that participating in sports regularly can improve personal confidence in social activities, learning to control one’s emotions while improving one’s interpersonal relationships (Qin and Qin, 2023). It can be seen that better emotional control and good partnerships can reduce the probability that children will bully others. Good peer relationships can help them when they experience bullying, but in contrast, isolated children are more likely to be bullied (Zhu et al., 2019). Both an analysis of data based on U.S. high school students by Nikolaou and Crispin (2022) and an 8-week experiment of preschool physical activity intervention by Hormazábal-Aguayo et al. (2019) further suggest that prolonged physical activity can be effective in reducing the likelihood of bullying among children. Based on the analysis of the above studies, sports participation has a substantial impact on bullying in schools. On this basis, we propose the following hypothesis:

*H1*: Sports participation plays an influential role in children’s perpetration of bullying.

*H2*: Sports participation plays an influential role in children’s exposure to bullying.

### 1.2 The mediating role of mental toughness

Mental toughness is the ability to bring people back to primary equilibrium or to a higher level of equilibrium psychologically and to successfully adapt to life under threatening conditions. In other words, resilience is an individual’s ability or trait to cope with stress, frustration, and injury (Davydov et al., 2010). Specifically, mental toughness can be viewed as a dynamic process in which a combination of stressful and protective factors lead to a well-adjusted individual (Lazarus, 1993). This is also supported by the conclusion that setbacks and losses suffered during sport participation contribute to the development of children’s mental toughness, as suggested in the study by Murphy et al. (2022). Belcher et al. (2021) found that sport participation positively affects brain structure and function, leading to better top-down self-regulation and contributing to the psychological resilience of adolescents affected by psychological problems. Therefore, we can find that children’s psychological resilience can be better improved and stress reduction positively achieved through sports participation (Whiter and Bennie, 2015). Whereas the emergence of bullying in schools reflects the physical and psychological gap between the perpetrators and the bullied (Olweus, 1994). Research has shown that children who experience bullying in schools have lower levels of individual mental toughness compared to children who have a non-traumatized childhood. This affects their own levels of physical and mental toughness in adulthood as well (Ross et al., 2020). It is further supported by the study of Hinduja and Patchin (2017), who concluded that mental toughness has become an important protective factor against bullying in schools and that students with higher levels of mental toughness are less likely to be affected by bullying in schools and that higher levels of mental toughness may also prevent bullying or reduce the negative effects of bullying. In the existing research, there is a significant negative correlation between school bullying and mental toughness (Pei, 2024). In a study of medical students, it was found that psychological toughness has a protective effect on the individual’s tendency to conduct problems, and students with high psychological toughness can better comply with social moral norms in adversity and setbacks, thus reducing the occurrence of bullying behavior (Jia and Chen, 2018). Narayanan and Betts (2014) took 393 students as research objects and studied the relationship between their psychological resilience and bullying behavior, and the results showed that enhancing students’ psychological resilience would reduce the occurrence of bullying behavior. Therefore, increasing children’s mental toughness will not only reduce their own behaviors of bullying and being bullied, but will also have a positive impact on children’s psychological status (Narayanan and Betts, 2014). Víllora et al. (2020) also provide evidence that children with lower levels of mental toughness are more likely to be bullied in school. These findings suggest that there is a significant relationship between children’s mental toughness and bullying in schools, and that mental toughness can be viewed as a result of an individual’s behavioral and psychological state in response to negative situations (Luthar et al., 2000). Specifically, high levels of sports participation can improve people’s physical structure and thus help support them in their mental struggle against adversity. As a result, children with high levels of mental toughness may further reduce their risk of bullying in schools and may be more effective in avoiding bullying. Therefore, the following hypotheses were developed:

*H3*: Mental toughness mediates the relationship between sport participation and perpetrated bullying.

*H4*: Mental toughness mediates the relationship between sports participation and exposure to bullying.

**Figure 1:**
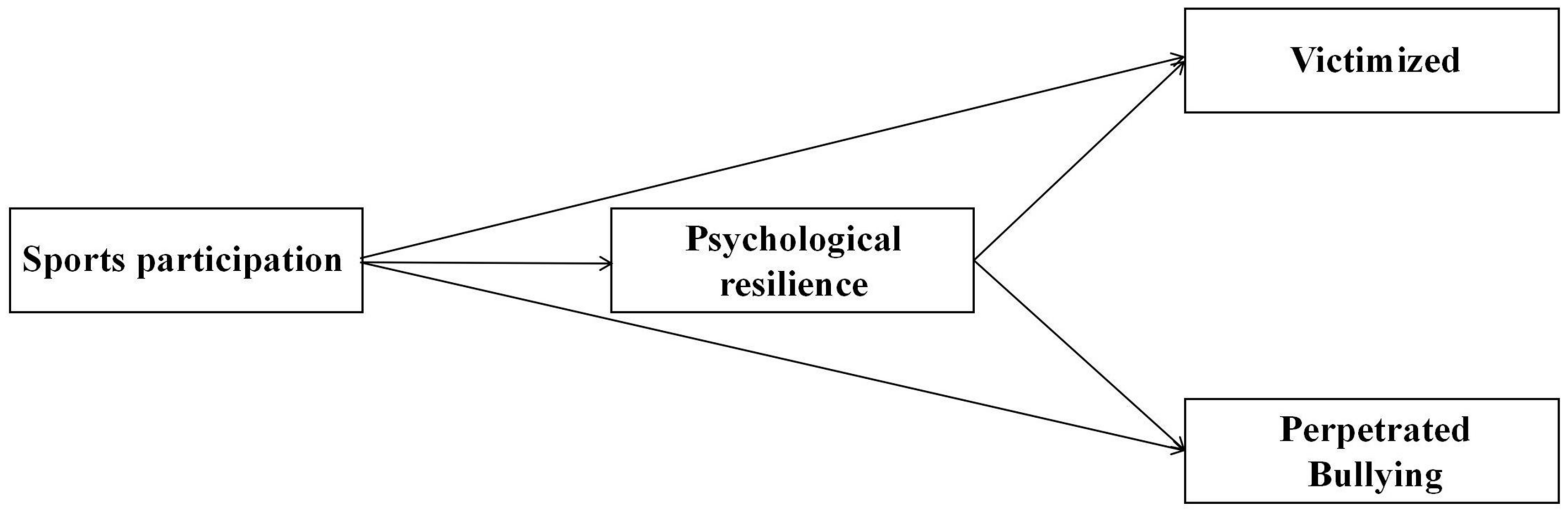
The proposed theoretical model.

In summary, children with high levels of sports participation are more likely to use sports participation as a way to de-stress and reduce stress, thereby gradually increasing their levels of mental toughness. This higher level of mental toughness provides them with more ways to cope with and avoid bullying in schools. Conversely, children’s lower levels of sports participation may result in lower levels of their own mental toughness, preventing such factors from serving to protect children and reduce their risk of school bullying. Based on these, the present study examines sport participation and the potential impact on children’s school bullying. In addition, this study examines the mediating role of mental toughness in the relationship between sport participation and school bullying. Based on these analyses above, we propose the following model (Figure 1).

## 2 Materials and methods

### 2.1 Sampling and procedures

This research is a cross-sectional survey and the target institutions are selected by means of simple random sampling. Firstly, subjective factors were excluded and the schools were classified according to administrative boundaries. Secondly, in order to ensure the validity and reliability of the research results, the sample size was calculated using the sample size calculation formula, and the sample size was calculated according to Equation 1. Finally, three schools in the city and five classes in each of the two rural schools were randomly selected proportionally for questionnaire survey.

Furthermore, prior to conducting the questionnaire survey, this study obtained an ethical approval exemption certificate in accordance with relevant policies.


(1) $$N=\frac{Z^{2}\;p\left(1-p\right)}{E^{2}}$$

In this study, 861 students were randomly selected as participants from five elementary school in Shandong Province, China. And the ages of the participants were 9–12 years old. On the premise of obtaining the consent of the participants, the participants were asked to fill in the paper version of the questionnaire by the way of on-site investigation, and demographic information about the sex and place of residence of the participants was collected. After excluding invalid questionnaires with missing answers or consistent responses, 712 questionnaires were collected with a response rate of 82%. Among them, 353 were boys and 359 were girls. Valid respondents included elementary school students from grade 3 to grade 5, with 618 living in urban areas and 94 in rural areas.

Inclusion criteria: (1) Primary school children aged 9–12 years; (2) Clear cognition, no expression disorder; (3) Informed consent, voluntary participation in this investigation.

Exclusion criteria: primary school children who have communication difficulties or do not agree to participate.

After data processing, the collected questionnaires are used for descriptive analysis, correlation analysis and mediation test. This is done using SPSS 24.0, SPSSprocess 4.1, and AMOS.

### 2.2 Questionnaire design

#### 2.2.1 Sports participation scale

Sports participation can be classified into direct sports participation and indirect sports participation. Among them, direct sports participation refers to people’s personal involvement in sports activities, such as physical exercise. Indirect sports participation means having a certain degree of love and interest in sports activities, but participating in them only as an audience or consumer, such as watching sports competitions and making sports consumption (Qiu et al., 2008). Through existing research, it has been found that the studies on sports participation mainly focus on active sports participation (Guo and Yu, 2022; Ouyang et al., 2020). Moreover, for children, direct sports participation is more sensitive to the development of their own psychological resilience. Therefore, based on the relevant research results and the research focus of this article, we will mainly focus on children’s direct sports participation, that is, by measuring the frequency, items, intensity and duration of children’s participation in sports activities to calculate the level of children’s sports participation. Thus, we choose the physical activity scale to assess the differences in sports participation levels among children.

The Physical Activity Rating Scale (PARS-3) used in this study has been previously used to measure sports participation (Duan et al., 2022). This scale was developed by Japanese scholar Hashimoto and revised by Chinese scholar Liang (1994) to investigate sports participation from three dimensions: intensity, duration and frequency of physical exercise (Liang, 1994), is designed to investigate the three dimensions of physical activity intensity, duration of a single exercise session, and the number of exercise sessions of the subjects, with one question for each dimension, all of which are scored on a scale of 1 to 5. According to the criteria of the scale, physical participation = physical exercise intensity × (physical exercise duration − 1) × physical exercise frequency, and the interval of physical exercise score is 0–100, which can be divided into three bands to indicate different levels of physical exercise: low exercise level is ≤19 points, medium exercise level is 20–42 points, and high exercise level is ≥43 points. The Cronbach’s *α* coefficient of this scale in this study was 0.795, KMO statistic was 0.681. According to the data, *α* ≥ 0.5, KMO ≥ 0.6，exist statistical significance, which can be further studied.

#### 2.2.2 Mental toughness scale

A psychological resilience scale that can measure the dimensions of emotional control and interpersonal assistance was developed by Chinese scholars Hu et al. (2008) through a combination of interviews and questionnaires. The scale is divided into five dimensions: goal focus, emotional control, positive cognition, family support, and interpersonal assistance, with 27 items, of which goal focus, emotional control, and positive cognition are human factors. While family support and interpersonal assistance are supportive factors, e.g., “After experiencing setbacks, I generally become more mature and experienced.,” “When I encounter difficulties and need assistance, I usually become more mature and experienced.,” “When I am in trouble and need help, I do not know who to go to.” The Likert five-point scale was used, with scores ranging from 1 to 5 indicating “not at all” to “completely,” with higher scores indicating greater mental toughness, and the scale has good reliability and validity. The Cronbach’s *α* coefficient of this scale in this study was 0.868, KMO statistic was 0.917, *χ*^2^/df = 7.76, IFI = 0.711, CFI = 0.709, TLI = 0.675, GFI = 0.762, AGFI = 0.714 and RMSEA = 0.098. According to the data, *α* ≥ 0.5, KMO ≥ 0.6, and RMSEA ≤ 0.1, exist statistical significance, which can be further studied.

#### 2.2.3 School bullying scale

This scale is the Chinese version of the Olweus Bullying Questionnaire (OBQ), revised by Chinese scholar Zhang Wenxin, which measures “bullying” and “being bullied” respectively (Olweus, 1994; Zhang and Wu, 1999). The questionnaire consists of a “Bullying Behavior Scale” and a “Being Bullied Behavior Scale,” with 12 items, such as “People give me unpleasant nicknames, people call me names, or make fun of and satirize me. “I purposely kept one or more of my classmates out of certain activities, excluded them from my friends, or made my friends ignore him/her completely.” The main measure of the respondents’ bullying in the past 12 months in the dimensions of physical bullying, verbal bullying and relational bullying. The scale is scored on a 5-point scale, with scores ranging from 0 to 4 indicating “never” to “several times a week,” 0 indicating no bullying or no being bullied, 1 and above indicating bullying or being bullied, and higher scores indicating more frequent bullying or being bullied. The higher the score, the more frequent the bullying or being bullied was practiced. The scale has good reliability and validity. The Cronbach’s alpha coefficient for the scale in this study was 0.951, KMO statistic was 0.961, *χ*^2^/df = 7.779, IFI = 0.946, CFI = 0.946, TLI = 0.933, GFI = 0.909, AGFI = 0.865 and RMSEA = 0.098. According to the data, *α* ≥ 0.5, KMO ≥ 0.6, and RMSEA≤0.1, exist statistical significance, which can be further studied.

### 2.3 Statistical analysis

Statistical analyses were performed using SPSS 24.0, SPSSprocess 4.1 and AMOS 27. We used SPSS 24.0 for descriptive analysis and correlation analysis, used SPSSprocess 4.1 for mediation test, and finally used AMOS to build a structural equation model to check the fit degree of the model. (1) Statistical tests for common method bias were conducted using Harman one-way factor analysis. All items of sports participation, psychological resilience, and school bullying were extracted and included in the exploratory factor analysis, and the results showed that there were eight factors with eigenvalues greater than 1 when non-rotated. And the explanation rate of the first factor was 26.008%, which was far below the critical criterion of 40%, indicating that there was no serious common method bias in this study. (2) Descriptive statistical analyses and Pearson correlation analyses were conducted for sports participation, mental toughness, and bullying in schools. (3) SPSSprocess 4.1 was used to conduct the mediation test, and the bootstrap procedure for this study was based on 5,000 samples, and 95% confidence intervals were generated to test the significance of the indirect effects. When the *p*-value is less than 0.05, it indicates that the content under study is statistically significant (Sun et al., 2025). Therefore, in this study, the p-value threshold was set at three levels: 0.05, 0.01 and 0.001, in order to verify whether the data have statistical significance.

## 3 Results

### 3.1 Descriptive statistics and correlation analysis

According to Table 1, there were 353 male participants (49.6%) and 359 female participants (50.4%) in this study. Six hundred eighteen people (86.8%) lived in urban areas and 94 people (13.2%) lived in rural areas.

**Table 1:** Demographic table of participants (*N* = 712).

Variable	Options	Frequency	Percent	*M*	SD
Sex	Male	353	49.6%	1.5	0.5
	Female	359	50.4%		
Place of residence	Town	618	86.8%	1.13	0.339
	Countryside	94	13.2%		

The mean and standard deviation of the variables and the results of the correlation matrix between the variables are detailed in Table 2.

**Table 2:** Descriptive statistics and correlation analysis (*N* = 712).

	*M*	SD	1	2	3	4
1 Sports participation	23.04	21.034	1			
2 Mental toughness	2.979	0.459	0.078*	1		
3 Perpetrated bullying	2.05	3.541	−0.131**	−0.206**	1	
4 Victimized	3.12	3.567	−0.076*	−0.147**	0.774**	1

As shown, *p < 0.05, **p < 0.01.

As shown in Table 2, the data supported their path. In particular, sports participation and child perpetration of bullying established a significant negative correlation (*r* = −0.131, *p* < 0.01); sports participation and child exposure to bullying established a significant negative correlation (*r* = −0.076, *p* < 0.05); sports participation and mental toughness established a significant positive correlation (*r* = 0.078, *p* < 0.05); mental toughness and child perpetration of bullying established a significant negative correlation (*r* = −0.206, *p* < 0.01); and mental toughness and child perpetration of being bullied established a significant negative correlation (*r* = −0.147, *p* < 0.01). The relationships between these variables supported the subsequent hypothesis testing.

### 3.2 Tests of mediation effects

To analyze the mediation effects, we used SPSSprocess 4.1 to test the mediation effects with a sample size of 5,000 and a confidence level of 95%. The model was fitted using AMOS and the model fit indicators were *χ*^2^/df = 6.837, IFI = 0.925, CFI = 0.925, TLI = 0.911, GFI = 0.898, AGFI = 0.86 and RMSEA = 0.091. Each of the above indicators were within acceptable limits, proving that the model was ideal.

Significance tests for mediating effects were conducted using a non-parametric percentage bootstrap procedure with 5,000 replicates and 95% confidence intervals calculated. Accordingly, we analyzed the results of the mediating effect of mental toughness between sports participation and children’s perpetration of bullying and exposure to bullying (see Tables 3, 4 for more details). In the mediating effect of sports participation, mental toughness, and perpetration of bullying, the value of the total effect was −0.0223, and the direct effect of sports participation on children’s perpetration of bullying was −0.0195, with a 95% confidence interval of [−0.0317, −0.0074],which does not contain 0. The direct effect is significant and hypothesis 1 is valid. The indirect mediating effect size for mental toughness was −0.0027, 95% confidence interval was [−0.0053, −0.0004], which did not contain 0, suggesting that there is a significant mediating effect of mental toughness between sport participation and child perpetration of bullying, hypothesis 3 was established. In the mediating effects of sports participation, mental toughness, and exposure to bullying, the total effect value is −0.0134, and the direct effect of sports participation on children’s exposure to bullying is −0.0115, with a 95% confidence interval of [−0.0239, 0.001], which contains 0. Therefore, the direct effect is not significant, and hypothesis 2 is not established. However, the indirect mediating effect of mental toughness was −0.002, with a 95% confidence interval of [−0.0038, −0.0003], which did not contain 0, indicating that there is a significant mediating effect of mental toughness between sports participation and children’s exposure to bullying, and hypothesis 4 is valid.

**Table 3:** Moderating effects between sports participation, mental toughness, and perpetration of bullying (*N* = 712).

	Effect	Boot SE	Boot LLCI	Boot ULCI
Total effect	−0.022	0.006	−0.035	−0.01
Direct effect	−0.02	0.006	−0.03 2	−0.00 8
Indirect effect	−0.003	0.001	−0.0053	−0.00 1

Boot SE, Boot LLCI, and Boot ULCL are the standard deviation, lower, and upper bounds, respectively, of the 95% confidence intervals for the indirect effects estimated by the bias-corrected percentile bootstrap method.

**Table 4:** Moderating effects between sports participation, mental toughness, and exposure to bullying (*N* = 712).

	Effect	Boot SE	Boot LLCI	Boot ULCI
Total effect	−0.013	0.006	−0.026	−0.001
Direct effect	−0.012	0.0063	−0.024	0.001
Indirect effect	−0.002	0.0009	−0.004	−0.00 1

Boot SE, Boot LLCI, and Boot ULCL are the standard deviation, lower limit, and upper limit, respectively, of the 95% confidence intervals for the indirect effects estimated by the bias-corrected percentile bootstrap method.

**Figure 2:**
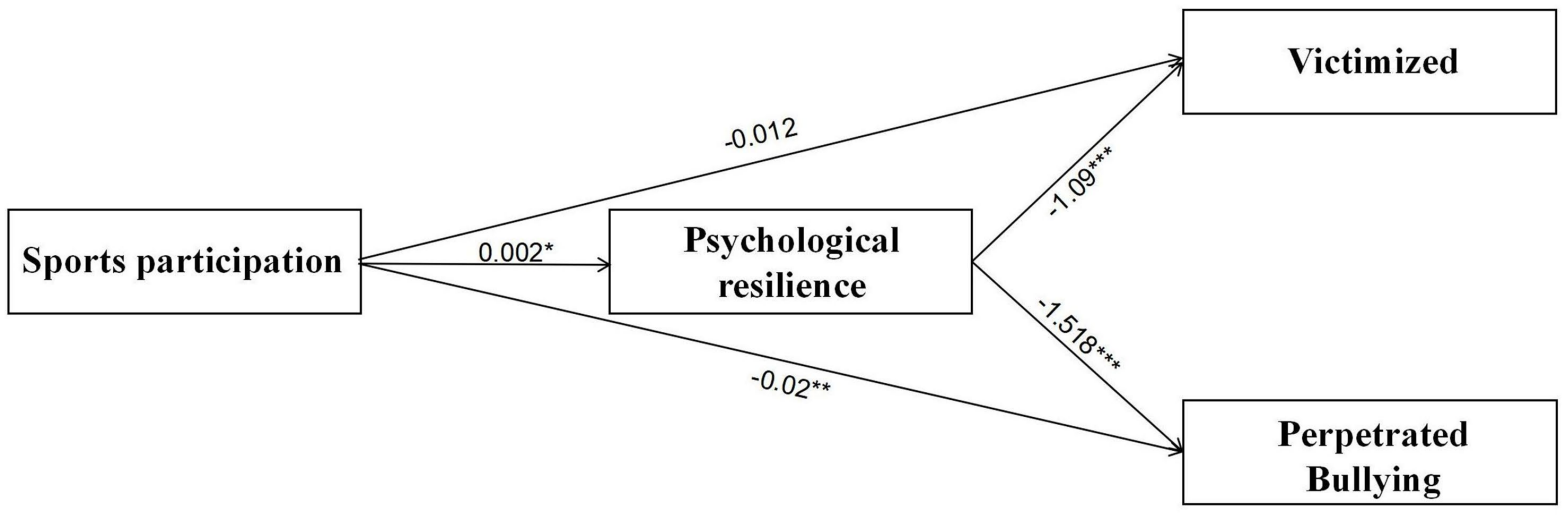
The mediation model. As shown, *p < 0.05, **p < 0.01, ***p < 0.001.

The specific pathways through which sport participation acts through mental toughness with children perpetrating bullying and children experiencing bullying are detailed in Figure 2 and Table 5.

**Table 5:** Model path relationship test (*N* = 712).

Path relation	Coeff	se	*t*	*p*
Sports participation → Psychological resilience	0.002	0.0008	2.2057	0.0277
Sports participation → Perpetrated bullying	−0.02	0.0062	−3.1541	0.0017
Psychological resilience → Perpetrated bullying	−1.518	0.283	−5.3631	0
Sports participation → Victimized	−0.012	0.0063	−1.8121	0.0704
Psychological resilience → Victimized	−1.09	0.2894	−3.7681	0.0002

According to Figure 2 and Table 5, all the path relationships of this study can be seen. (1) In the effect of Sports participation on Psychological resilience, coeff = 0.002, *p* < 0.05. (2) In the effect of Sports participation on Bullying, coeff = −0.02, *p* < 0.05. (3) In the effect of Psychological resilience on Bullying, coeff = −1.518, *p* < 0.001. (4) In the effect of Sports participation on Victimized, coeff = −0.012, *p* > 0.05. (5) In the effect of Psychological resilience on Victimized, coeff = −1.09, *p* < 0.001.

Differences between the two mediating effects were compared, and the data results are detailed in Table 2. In the pathways of sports participation, mental toughness, and child-perpetrated bullying, the 95% confidence intervals for the direct effects were [−0.0317, −0.0074], which did not include 0. The 95% confidence intervals for the indirect effects were [−0.0053, −0.0004], which also did not include 0. It suggested that mental toughness played a partial mediating role in this pathway. For the pathways of sports participation, mental toughness, and children’s exposure to bullying, the 95% confidence interval for the direct effect was [−0.0239, 0.001], which included 0. The 95% confidence interval for the indirect effect was [−0.0038, −0.0003], which did not include 0, suggesting that mental toughness played a full mediating role in this pathway.

## 4 Discussion

As an important influence on children’s physical and mental health, school bullying has an increasing impact on children’s healthy growth, and its research has received more and more attention. Bullying in childhood not only has a negative impact on one’s daily life, learning and psychology, but can even have an important negative impact on children’s future development (Ikeda et al., 2020). However, existing studies have mainly explored the prevention mechanism of bullying in children’s schools, and paid less attention to the influencing factors of children’s practicing and suffering from bullying. Therefore, this study constructed a model between sports participation, children’s mental toughness and school bullying to explore the effects and pathways of sports participation and mental toughness on children’s perpetration of bullying and exposure to bullying. The model helps us understand how and under what conditions sport participation influences children’s perpetration of bullying and exposure to bullying, providing potential guidance for reducing school bullying and improving children’s mental and physical health.

### 4.1 The relationship between sports participation and school bullying

#### 4.1.1 The relationship between sport participation and bullying perpetration

As shown in Table 1, the correlation between sports participation and perpetrating bullying is −0.131, which is negative correlated and significant. The results of the study showed that the effect size of sports participation in predicting children’s perpetration of bullying was −0.02 (Table 2). H1 is valid, which is consistent with the results of previous studies like (Qin and Qin, 2023). The predictive effect remained significant after the inclusion of mediating variables. That is, the lower a child’s level of sports participation, the more likely he or she is to develop the behavior of perpetrating bullying on others (Ji et al., 2004). This may be due to the fact that children with low levels of sports participation lack the necessary means to express their negative emotions and communicate with others, which not only makes children lack the necessary emotional output and communication skills when encountering problems or arguments (Qin and Qin, 2023), but also makes them more likely to feel stressed and may have a certain adverse effect on their psychology (Whiter and Bennie, 2015).

#### 4.1.2 The relationship between sports participation and exposure to bullying

As shown in Table 1, the correlation between sports participation and children’s exposure to bullying was −0.076, a negative and significant correlation. The results of the study showed that the effect size of sports participation in predicting children’s exposure to bullying was −0.012 (Table 3), with a non-significant direct effect and an invalid H2. But the prediction was significant with the inclusion of the mediating variables. This implies that the probability of children experiencing bullying cannot be directly reduced by increasing the level of sports participation, but interventions and influences can be implemented through the mediating role of mental toughness.

In summary, the results of this study further emphasize that sports participation significantly predicts children’s perpetration of bullying and also indirectly predicts children’s perpetration and exposure to bullying through mental toughness. The results of the study also further confirm the feasibility of sports participation to influence and reduce the incidence of bullying in schools.

### 4.2 The mediating role of mental toughness

The results of the study showed a significant positive correlation of 0.078 (Table 1) between sports participation and mental toughness. Mental toughness mediated the relationship between sports participation and children perpetrating bullying with an effect size of −0.003 (Table 2), with H3 being valid, and mental toughness also mediated the relationship between sports participation and children’s exposure to bullying with an effect size of −0.002, with H4 being valid. The study showed that children with high levels of sports participation had a good emotional output style, were less stressed themselves and more willing to communicate with others than children with low levels of sports participation, making them able to adjust their emotions at any time to cope with changes and pressures in the external environment. At the same time, more peers were willing to help them when they encountered difficulties, which improved their psychological resilience (Zhu et al., 2019; Víllora et al., 2020). The results of the present study also showed that the psychological resilience of the children was improved. The results of this study also showed a significant negative correlation between mental toughness and children’s perpetration of bullying, and between mental toughness and children’s exposure to bullying, which is consistent with previous studies (Pei, 2024). Mental toughness does play an important role in children’s perpetration or exposure to bullying, helping to reduce their stress and stabilize their emotional state, which has a significant impact on promoting children’s mental health development (Ross et al., 2020). For children, strong mental toughness allows them to seek solutions when they are bullied and to talk to their peers, teachers or parents to resolve the problem positively. Mental toughness also enables children to become strong enough to trust others, to be more understanding, tolerant and caring (Shaw et al., 2023), and to reduce the incidence of bullying of other children. In summary, sports participation can reduce the likelihood of children committing or experiencing bullying by increasing their mental toughness. This study analyzes the complex relationship between sports participation, mental toughness, and school bullying, which enriches the research related to children’s school bullying to some extent.

### 4.3 Theoretical and practical significance

This study has some theoretical and practical implications. From a theoretical perspective, the study enriches the literature on factors influencing bullying in children’s schools. Specifically, the study establishes a negative correlation between sport participation and children’s perpetration of bullying, emphasizing that higher levels of sport participation intensity are associated with a lower likelihood of children perpetrating school bullying. This enriches the literature on bullying in children’s schools. In addition, this study suggests that mental toughness can mediate the association between sport participation on children’s perpetration or exposure to bullying, contributing to the understanding of the mechanisms by which sport participation acts on bullying in children’s schools. Children with higher levels of sport participation can reduce bullying of others or reduce the likelihood of experiencing bullying by increasing their mental toughness.

From a practical point of view, this study provides directions for the prevention and reduction of bullying in schools for children in terms of physical activity and individual psychology. In terms of sports participation, schools should develop regular sports programs that meet the interests of students to increase children’s interest in physical activities and improve their level of sports participation. In terms of psychological resilience, teachers and parents should not only pay attention to students’ academic performance, but also pay attention to changes in the children’s daily performance, increase the frequency of communication with children in their daily lives, and then give them targeted support and help to improve their level of psychological resilience. At the same time, schools can offer courses related to physical and mental health for children every week to enhance their psychological resilience. Besides, schools can conduct regular physical health and mental health assessment of children, while checking children’s physical and mental health levels, screening children who may have committed or suffered from bullying for early intervention, in order to reduce the likelihood of bullying incidents in schools. On the one hand, in addition to fulfilling the tasks of the physical education curriculum, schools should design more extracurricular activities according to children’s interests and establish more incentives to encourage children to engage in a higher level of sports participation, so as to enhance children’s mental toughness through sports participation. On the other hand, parents should be good at communicating with their children and increase the amount of time they spend exercising with their children, which not only provides them with correct guidance and positive encouragement, but also enables them to identify their own physical and mental health problems in a timely manner, and continuously improves the level of children’s participation in sports and mental toughness. At the same time, children should also have clearer goals to increase their physical activity time, in order to maintain a stable mood and self-confidence to improve their mental toughness.

### 4.4 Limitations and future research directions

Although this study provided insights into the factors influencing sports participation and bullying in schools, there are some limitations in future research that could further improve the understanding of this area. As this study used children’s self-administered questionnaires, there are the following problems: (1) this study only used a cross-sectional study, which is a correlational study, and could not explore the causal relationship between the variables, and the continuous and stable relationship of the response variables. In the future, interviews could be conducted with teachers of some schools as well as children’s parents, to further explore the impact of sports participation on children’s bullying in schools, in order to improve the external validity of this study and verify the existing research findings. It is also possible to conduct longitudinal studies on children’s participation in sports, psychological resilience and school bullying in the future. This can provide more accurate data support for the application of sports participation in preventing and reducing school bullying. It can also enable people to have a clearer understanding of the changing trends of children’s sports participation and psychological resilience, as well as their relationships with school bullying. Furthermore, in future research, it is possible to explore the influence of various activities on children’s psychological resilience, in order to investigate whether they can also contribute to the enhancement of children’s psychological resilience. (2) The sample of this study was taken from only five elementary schools in one province in northern China, and the ecological validity of the findings may be low. Therefore, the results of this study cannot be easily generalized to geographic areas with other environmental differences and cultural backgrounds. In the future, the sample size can be enlarged for revalidation or adding research studies in other cultural contexts. Furthermore, verifying the results of these issues in other cultures and geographical contexts will be of great help to this research. (3) There are many other factors that can influence children’s bullying in schools, such as family upbringing styles, subjective well-being, et al., and more variables can be used in future studies to produce more comprehensive and meaningful results. Furthermore, these variables can be incorporated into the model of this research. By exploring the interactions and joint effects of various factors, a more comprehensive understanding of the influences and complex nature of the phenomenon of bullying in children’s schools can be achieved. (4) This study merely analyzed and demonstrated whether participation in sports and psychological resilience could influence students’ bullying behaviors on campus. It did not conduct further research on how participation in sports could affect children’s bullying behaviors on campus or which type of sports participation could better reduce such behaviors. Firstly, in future studies, longitudinal research over a long period could be conducted to verify the various possible impacts of sports participation on becoming a bully or a victim. Secondly, the types and projects of sports participation could be further refined to analyze more clearly and precisely which sports participation methods could have a more effective impact on reducing children’s bullying behaviors. (5) This study only examines the behaviors of bullying and verbal bullying among students in the context of school bullying, but does not cover the aspect of cyberbullying. Considering the different natures of data acquisition, future research can include cyberbullying in the research scope to further demonstrate the impact of various bullying behaviors on students and make the research results more accurate and valuable.

## Data Availability

The original contributions presented in the study are included in the article/Supplementary material, and further inquiries can be directed to the corresponding author/s.
